# Modern contraceptive use among postpartum women living with HIV attending mother baby care points in Kabarole District, Uganda

**DOI:** 10.1186/s12905-020-00944-4

**Published:** 2020-04-22

**Authors:** Andrew K. Tusubira, Simon Peter Sebina Kibira, Fredrick Edward Makumbi

**Affiliations:** 1 Department of Health Policy, Planning and Management, School of Public Health, College of Health Sciences, Makerere University, P. O Box 7072, Kampala, Uganda; 2 Department of Community Health and Behavioural Sciences, School of Public Health, College of Health Sciences, Makerere University, Kampala, Uganda; 3 Centre for International Health, Department of Global Public Health and Primary care, Faculty of Medicine, University of Bergen, Bergen, Norway; 4 Department of Epidemiology and Biostatistics, School of Public Health, College of Health Sciences, Makerere University, Kampala, Uganda

**Keywords:** Contraception, Family planning, Unmet need, Antenatal, Postpartum, HIV, Uganda

## Abstract

**Background:**

Preventing unintended pregnancies among women living with HIV is important for improving maternal and child health outcomes. Despite multiple contacts between postpartum women and healthcare providers at health facilities, modern contraceptive use during postpartum period remains low even among women with intentions to limit or delay childbearing. We estimated postpartum modern contraceptive use, unmet need and factors associated with modern contraceptive use among HIV positive women attending mother-baby HIV care points.

**Methods:**

We conducted a cross-sectional study, between April and May 2016, among HIV positive women attending postpartum care at six health facilities in Kabarole district, Uganda. Health facilities were stratified by level prior to selecting participants using systematic sampling. We administered structured questionnaires to women who had delivered within the last two to 18 months. Women who reported current use of a modern method were categorized as modern contraceptive users. Women not using but wanted to stop childbearing or space childbirth by at least 2 years were considered to have unmet need for modern contraception. We estimated a modified Poisson regression model to examine variations in the use of modern methods by various characteristics of participants.

**Results:**

We interviewed 369 women. Forty percent of them were using a modern method, with injectables being the most widely used. A third (33%) of the women had unmet need for modern methods, with unmet need for spacing (24%) being higher than for limiting births (9%). Modern contraceptive use was significantly higher among women who had delivered seven or more months earlier (Adj.PR = 2.02; CI: 1.49, 2.74); women who were counseled on family planning during antenatal care (Adj.PR = 1.53; CI: 1.07, 2.18); those who obtained methods through the care points (Adj.PR =2.27; CI: 1.32, 3.90); and those who jointly made decisions regarding childbearing with their partners (Adj.PR = 1.49; CI: 1.02, 2.17).

**Conclusion:**

Use of modern contraceptives was low while unmet need was high among postpartum women living with HIV. The findings suggest that there are gaps which indicate the need to strengthen contraceptive service delivery at these care-points. Providing family planning counseling early would improve uptake of contraception upon resumption of menses and sexual activity.

## Background

Unintended pregnancies refer to pregnancies that are either unwanted (no additional child is desired by women or couple) or mistimed (when the pregnancy occurs earlier than desired) [1]. Globally, it is estimated that 40% of all pregnancies among women are unintended [2]. Studies in sub-Saharan Africa show that unintended pregnancy is higher among women living with HIV than among HIV-negative women [3, 4]. Some studies in the region show that women living with HIV often resume or increase sexual activity as their health improves, and some engage in risky sexual behavior that result in unintended pregnancies [5, 6]. Uganda is one of the countries in sub-Saharan Africa with high prevalence of unintended pregnancy among women living with HIV. Estimates, for instance, show that nearly half (43%) of pregnancies among women living with HIV in the country are unintended [7], which is higher than some of the countries in the region, including Nigeria (37.2%) [8], Zimbabwe (35.1%) [4], and DR Congo (22%) [9]. The high levels of unintended pregnancies among women living with HIV in SSA suggest the need for strategies to prevent or reduce their occurrence. Prevention of unintended pregnancies among women living with HIV is, in turn, important not only for improving maternal and child health but also for eliminating new pediatric HIV infections [10, 11].

One strategy to reduce unintended pregnancies is through promotion of effective use of modern contraceptive methods among postpartum women [12, 13]. For the postpartum women, contraceptive use supports them to limit or delay childbearing depending on their reproductive intentions [14]. However, few postpartum women in sub-Saharan Africa embrace contraception and those using contraceptives often depend on short acting or traditional methods [15, 16]. Studies in the region show that some postpartum women misinterpret breastfeeding as a method of contraception even when the criteria for lactation amenorrhea method (LAM) are not met [17, 18]. To meet the criteria of LAM, a woman must be amenorrhoeic, exclusively breastfeeding, not menstruating, and within 6 months following a delivery. If any of these criteria is not met, the woman is no longer protected from pregnancy [19]. For example, a study in Egypt found that 25.3% of breastfeeding women had conceived, with 15.1% conceiving before return of menses [20]. In Uganda, postpartum contraception is low even among postpartum women who had multiple encounters with health facilities [21].

In 2013, mother-baby care points were established at health facilities in Uganda as specialized postpartum HIV care clinics. They were established with the aim of reducing mother-to-child HIV transmission to less than 5% through improved retention of both the HIV-positive woman and her exposed baby at the same care points [22]. The mother – baby pair are enrolled and retained in care for a period of 18 months and transfer from one facility to another is minimized. These clinics provide routine postpartum services including general examination and treatment of postpartum women, immunization, growth monitoring, infant and young child feeding support and family planning services. These services are typically integrated with antiretroviral therapy (ART) services such as early infant diagnosis, ART for infected infants and medicine refill for the woman [22, 23].

Enrolment into care at a mother baby care point is an opportune time to assess a woman’s fertility plans and intervene to prevent unintended pregnancies. Although modern contraceptives are effective and reliable among postpartum women living with HIV, there is limited understanding of the use of modern contraception among postpartum women living with HIV who are enrolled in these HIV-postpartum care points. Although these women access integrated HIV and postpartum care for 18 months, there is no documentation of the extent to which they take up contraception. Besides, it is also crucial to understand whether these women desire to limit childbirth or space their next birth for at least 24 months before conceiving again, and whether they have unmet need for modern contraception. Unmet need is a relevant reproductive health indicator for measuring the gap between women’s reproductive desire to avoid pregnancy and their contraceptive behavior (utilization gaps). It is also useful for program planning and evaluation. The Uganda demographic health survey data on unmet does not provide insights on the unmet need for modern contraceptive among postpartum women living with HIV. In this paper, we examine the extent of use of modern contraceptive methods, unmet need and factors associated with use of modern contraceptive methods among postpartum women living with HIV in Kabarole district, Western Uganda.

## Methods

### Study site

We conducted this study at mother baby care points at health facilities in Kabarole district. The district was purposively selected because it is located in a region with low modern contraceptive prevalence among married women of reproductive age in general (25%), [24] and those living with HIV in particular (22%) [25]. The HIV prevalence in this district was 16.1% among male and female adults [26], which is higher than the estimated HIV prevalence for adults in the other districts in the same region, which ranges between 5.7 and 7.9%, [27]. HIV prevalence among children aged 18 months or younger is 7% [28]. Uganda’s healthcare system is referral based with health facilities at different levels [29]. Each level of facility provides a recommended minimum healthcare package and refers cases beyond its scope of service to a higher level. Health center (HC) IIs are the lowest level, followed by HC level III, HC level IV and hospitals in that order. There were 30 level II HCs, 20 level III HCs, three level IV HCs and three hospitals in Kabarole. Every HC-III, HC-IV, and hospital had a mother baby care point.

### Study population

The study was among postpartum women living with HIV attending care at the mother-baby care points. We included postpartum women who had delivered in the past two to 18 months prior to the study. Only women with at least one postpartum care visit were included to allow for capturing of information on the services they received during the visits. We excluded those who had come to the care point seeking immediate care or attention from the healthcare workers, women who had become pregnant again after the previous delivery and those attending the mother-baby care points for the first time.

### Study design and sampling

We conducted a quantitative cross sectional study with a sample size estimated using the Kish- Leslie formula [30] under the following assumptions; 20% modern contraceptive use among postpartum HIV-positive women [31], a 5% level of precision in the estimate of modern contraceptive use and 95% confidence intervals. This gave a sample size of 246 women which was inflated by a factor of 1.5 [32], to account for clustering of individuals identified from the same health facility, resulting in a final sample size of 369 women [33].

Health facilities with mother-baby care points were stratified according to their level: health center IIIs, HC IVs and hospitals. Six high volume health facilities were randomly selected, two from each stratum; HC-III (Mugusu, Rwiimi), HC-IV (Kibito, Bukuku), Hospital (Kabarole and Fort portal regional referral hospital). The number of participants selected from each facility was determined based on probability proportional to size. The number of mother-baby pairs expected to be in care at these points was about 632 at the time of data collection. The sample size for each facility was proportionately allocated based on the volume of mother-baby pairs at the facility. This resulted in a sample size of 34 women from Rwimi H/C III, 37 from Mugusu H/C III, 52 from Bukuku H/C IV, 58 from Kibito H/C IV, 144 from Fort Portal hospital and 44 from Kabarole Hospital. In every selected health facility, study participants were selected using systematic random sampling. The number of mothers expected to attend a care point on a given clinic day was obtained from the appointment registers. This number was used to calculate the sampling interval. The sampling interval therefore differed each clinic day in each facility and across facilities. After selecting a woman, the interviewer explained the purpose of the study and screened women for study eligibility. Only eligible women who provided written informed consent were included in the study. All interviews were conducted prior to receipt of routine healthcare.

### Data collection

Eight trained research assistants with nursing background collected the data under the supervision of the first author in April and May 2016. The research assistants were not part of the staff at the health facility where data were collected. We used a pretested structured questionnaire adapted from UDHS 2011 [34] and PMA2020 [24] which was translated into *Rutooro* (the local language). Research assistants administered face-to-face interviews in private rooms, in the health facilities, in either English or the local language (Rutooro).

### Study variables and measurements

The outcome variable was “current use of a modern contraceptive method”, defined as any participant who was using a modern contraceptive method at the time of the study. Women who were using any modern method were coded 1 and non-users 0. Modern contraceptive methods considered were, pills, injectables, condoms, implants, intrauterine devices and systems (IUD/ IUS) and permanent methods (female or male sterilization) [35]. Participants who reported using condoms were asked about the frequency of use, (either sometimes or always), every time they had sexual intercourse since giving birth. Only those who reported always using condoms (consistent use) for each sexual encounter, irrespective of the partner, were considered as contraceptive users. Unmet need for contraception was defined as the percentage of fecund and sexually active women who were married or in consensual union and who reported not wanting any more children or wanting to delay their next child birth by at least 2 years or were unsure when to have a child but were not using a modern contraceptive method at the time of the survey [36]. Sexually active women were those who reported resumption of sexual intercourse after recent childbirth. Unmet need was categorized into unmet need for limiting, for those who did not want any more child; and unmet need for spacing for those who wanted to delay their next birth by at least 2 years or were unsure about their preferred timing for next birth but were not using a modern contraceptive method.

The study was informed by Anderson’s 1995 model of health care utilization [37]. The model consists of three major constructs: predisposing, enabling and need factors. Predisposing factors were the socio-demographic characteristics of the women. Enabling factors comprised family support (i.e., male partner related factors) and health service factors. Need factors included fertility and health related indicators. Indicators under each construct were based on existing literature including studies on modern contraceptive use among People Living with HIV (PLWHIV) [7, 38], Uganda’s demographic and health survey [34] and ongoing reproductive health surveys in the country [24].

Data were captured on women’s socio-demographic characteristics, reproduction and fertility including age, education level, period since childbirth, fertility desire, parity, return of menses and resumption of sexual activity. Period since child birth was categorized into 2–3, 4–6 and more than 6 months, following the three phases of postpartum period which include: subacute postpartum period which starts 2 weeks after delivery; delayed postpartum period which lasts up to 6 months; and the postpartum period which is more than 6 months after delivery and comprises the extended postpartum period [39]. Other independent variables included women’s health-related factors (i.e., period since HIV diagnosis, use of ART and duration on ART), health service factors (number of times a woman had attended the care points, obtaining contraceptives at the care points and whether the woman had been counseled on family planning during antenatal care (ANC), after delivery, and at the mother-baby care points). Male partner characteristics included partner’s age, education level, fertility desire, HIV status, need of partner approval to use a contraceptive method, partner intention to use a male condom, and couple decision making on childbearing. A male partner was defined as a man who was in a marital or consensual union with the postpartum woman. In the study, all partners were male and there were no women reporting same-sex partners. Information regarding male partners was obtained from the women.

### Data analysis

Our analysis entailed generating descriptive statistics, cross-tabulations with Chi-square tests, and estimation of multivariable Modified Poisson regression models.

**Fig. 1:**
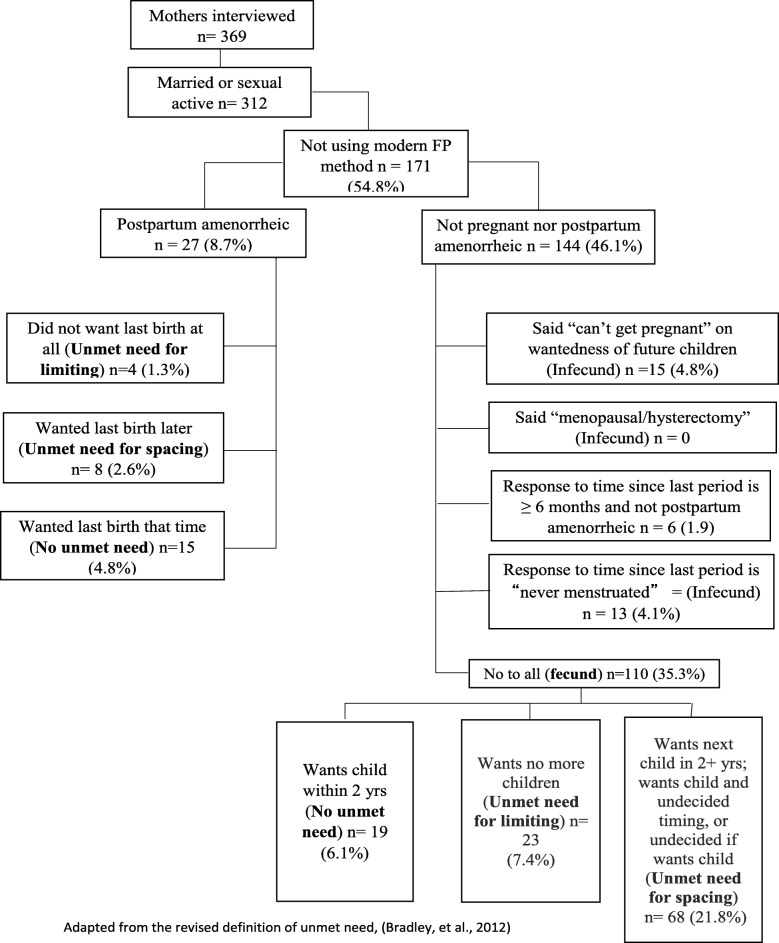
Unmet need among mothers living with HIV attending mother baby care points in Kabarole, Uganda

Descriptive analyses were conducted to obtain frequencies and percentages for the socio-demographic characteristics and the indicators of health and services uptake. We also obtained the mean and standard deviation for the women’s age. Unmet need for contraception was estimated using the revised algorithm for measuring such need from demographic and health surveys data [36]. The algorithm followed is presented in Fig. 1.

We conducted chi-square tests to determine the significance of association between the outcome variable (current use of a modern contraceptive method) and level of health facility. The purpose was to determine whether there were significant variations in the use of modern contraceptives by health facility. We estimated a multivariable modified Poisson regression model to determine adjusted prevalence ratios (PR), with 95% confidence intervals, of factors associated with use of modern contraceptive methods among women attending mother baby care points [40]. We obtained prevalence ratios instead of odds ratios because the dependent variable had a prevalence of more than 10% (at 39.8%). The literature suggests that using Odds Ratios (OR) when the outcome is higher than 10% tends to overestimate the strength of association [41, 42]. We estimated a modified Poisson regression model via generalized linear modeling with family (Poisson), link (log) and robust standard errors [43]. We further estimated a multivariate regression model that only included women who were married or in consensual union to determine the association between use of modern contraceptive methods and partner characteristics. We conducted likelihood ratio tests to identify the best fitting model. Adjusted prevalence ratios with *p*-value < 0.05 were considered statistically significant.

## Results

A total of 369 HIV positive postpartum women who were enrolled in care at mother-baby care points in Kabarole district were interviewed. Women were recruited from six health facilities with 51.2% being from hospitals, 30.1% from HC-IVs and 18.7% from HC-IIIs. All the 369 women who were requested to participate in the study and were eligible, accepted to be interviewed.

The average age of respondents was 29.7 (standard deviation = ± 6.3) years, with a range of 16–48 years. Forty six percent of the women had given birth within the past 6 months and the mean age of all the babies was 8.3 months (standard deviation = ±4.9). Fifty seven women (15.7%) had not attained any formal education and 52.6% had attained only primary level education. Majority (71.8%) were married or in a consensual union with their male partners at the time of the study being the fathers of their babies. More than half (62.3%) of the women were subsistence farmers. Forty-six percent had one or two living children, 56.9% did not want any more children, and two-thirds of the births were unintended (36% mistimed, 30.9% unwanted) (Table 1).

**Table 1:** Socio-demographic characteristics of women living with HIV attending mother baby care points, in Kabarole, Uganda

	Frequency	Percent
**Total**	369	100
**Age group**		
16–19	16	4.3
20–24	57	15.5
25–34	204	55.3
35–49	92	24.9
**Education level**		
None	57	15.4
Primary	194	52.6
Secondary	103	27.9
Tertiary	15	4.1
**Religion**		
Catholic	172	46.6
Protestant	110	29.9
Muslim	40	10.8
Other	47	12.7
**Marital status**		
Unmarried	104	28.2
Married or in consensual union	265	71.8
**Occupation**		
Farmer/peasant	230	62.3
Business woman	88	23.9
Salaried-job	36	9.8
Other	15	4.0
**Period since child birth**		
2 to 3 months	74	20.1
4 to 6 months	96	26.0
≥ 7 months	199	53.9
**Wanted the pregnancy**		
Wanted then	122	33.1
Wanted later	133	36.0
Not at all	114	30.9
**Number of living children**		
1–2	171	46.3
3–4	122	33.1
5 and above	76	20.6
**Desire for more children**		
No – more	210	56.9
Yes more	159	43.1

Less than half (48%) of the women had learnt that they were HIV positive within the last 3 years prior to the study. ART uptake was almost universal (98.4%), with majority (82.4%) starting ART treatment at least 1 year prior to the study. Three quarters of the respondents had attended the mother baby care points between two to six times (Table 2).

**Table 2:** Indicators of health and services uptake among women living with HIV attending mother baby care points in Kabarole, Uganda

	Frequency	Percent
**Total**	369	100
**Duration since HIV diagnosed (years)**		
Less than one year	35	9.5
–One year to two years	143	38.7
–three to four years	98	26.6
5 and more years	93	25.2
**On ARVs**		
No	6	1.6
Yes	363	98.4
**Period since started ARVs**		
less than a year	59	16
At least 1 year	304	82.4
Not on ARV	6	1.6
**Number of MBCP attendances**		
2 to 6 times	276	74.8
7 to 12 times	81	21.9
13 to18 times	12	3.3
**Previous use of modern contraceptives**		
No	90	24.4
Yes	279	75.6
**Fear of contraceptive side effects**		
No	222	60.2
Yes	147	39.8

*MBCP*Mother Baby Care point

### Use of modern methods and unmet need

Out of the 369 women interviewed, 39.8% were using a modern contraceptive method at the time of the study. There was no statistically significant association between use of modern contraceptive methods and the level of health facility a woman sought services from (*p* = 0.307). The most common modern methods were injectables (37.4%) and implants (32%), while female sterilization was the least used method (Table 3). Use of modern methods was highest among women who had given birth in the past four to 6 months (Prevalence =52%; 95% CI: 44.7, 58.7) and lowest among those who had given birth in the past two to 3 months (Prevalence =20%; 95% CI: 11.0, 29.5).

**Table 3:** Modern contraceptive methods mix among women living with HIV attending mother baby care points

Modern method used	Frequency	Percent^a^
Female sterilization	6	4.1%
Implants	47	32.0%
IUD	14	9.5%
Injectable	55	37.4%
Pills	13	8.8%
Male condom	29	19.7%

^a^Adds up to more than 100% because some participants were using multiple methods

A third (33%) of the women had unmet need for modern methods of contraception. There was higher unmet need for spacing than for limiting births (24.4 and 8.7%, respectively; Fig. 1).

### Factors associated with use of modern contraceptive methods

In the multivariable model, we adjusted for age group, period since child birth, FP counselling during ANC, obtaining contraceptives through the mother-baby care points, religion, period since HIV diagnosis, occupation, number of living children and desire for more children. Results showed that use of modern contraceptive methods was twice higher among women who had delivered seven or more months earlier compared to those who had recently delivered in past two to 3 months (Adj.PR = 2.02; 95% CI: 1.49, 2.74).

Modern contraceptive use was significantly higher among women who were counseled on family planning during ANC than among those who were not (adj.PR = 1.53; 95% CI: 1.07, 2.18) and among those who obtained contraceptives from the mother-baby care points than among those who did not (adj.PR = 2.27; 95% CI: 1.32, 3.90) (Table 4). The model involving only married women or those who were in consensual union adjusted for religion, occupation, number of living children, desire for more children, FP counselling during ANC, decision-making on childbearing, period since child birth, obtaining contraceptives through the mother-baby care points and male partner level of education. The results showed that modern contraceptive use was significantly higher among women who jointly made decisions regarding childbearing with their partners than among those who did not (adj.PR = 1.49; 95% CI: 1.02, 2.17) and among those who desired few children than among those who desired more children (adj.PR = 0.68, 95% CI: 0.51, 0.98) (Table 4).

**Table 4:** Prevalence ratios from multivariate Poisson regression models showing factors associated with modern contraceptive use among postpartum women living with HIV attending mother baby care points in Kabarole district, Uganda

Variable	Among all women	Married or in consensual union
	Adjusted PR (95% C.I)	Adjusted PR (95% C.I)
**Age group**		
16–19	1.0	
20–24	1.58 (0.65, 3.82)	
25–34	1.60 (0.69, 3.73)	
35–49	1.70 (0.71, 4.08)	
**Period since HIV diagnosis**		
< 1 year	1.0	
1–2 years	1.55 (0.88, 2.74)	
3–4 years	1.30 (0.71, 2.36)	
5 and more years	1.26 (0.69, 2.31)	
**Period since child birth**		
2 to 3 months	1.0	1.0
4 to 6 months	1.67 (0.21, 2.29)	1.85 (1.33, 2.57 ^a^)
≥ 7 months	2.02 (1.49, 2.74 ^a^)	1.75 (0.82, 2.54)
**Religion**		
Catholic	1.0	1.0
Protestants	1.94 (1.47, 2.56 ^a^)	2.16 (1.58, 2.96 ^a^)
Muslim	1.30 (0.99, 2.36)	1.60 (1.06, 2.41 ^a^)
Other	1.18 (0.69, 1.90)	1.47 (0.80, 2.71)
**FP counselling during ANC**		
No	1.0	1.0
Yes	1.53 (1.07, 2.18 ^a^)	1.41 (0.99, 2.02)
**Able to obtain contraceptives at MBCP**		
No	1.0	1.0
Yes	2.27 (1.32, 3.90 ^a^)	1.45 (0.82, 2.54)
**Occupation**		
Farmer/peasant	1.0	1.0
Business woman	0.96 (0.74, 1.23)	0.99 (0.76, 1.31)
Salaried-job	0.94 (0.59, 1.47)	0.92 (0.56, 1.52)
Other	0.78 (0.38, 1.59)	0.73 (0.25, 2.06)
**Number of living children**		
1–2	1.0	1.0
3–4	1.16 (0.87, 1.56)	0.97 (0.71, 1.32)
5 and above	0.86 (0.59, 1.26)	0.86 (0.61, 1.22)
**Desire for more children**		
No – more	1.0	1.0
Yes more	0.85 (0.65, 1.10)	0.68 (0.51, 0.98)
**Decision-making on childbearing**		
Male partner		1.0
Woman herself		1.12 (0.73, 1.73)
Joint agreement		1.49 (1.02, 2.17 ^a^)
**Partner attained education**		
Yes		1.0
No		1.01 (0.82, 2.55)

^a^*P*-value < 0.05 (significant), *PR* Prevalence ratio, *Freq* Frequency, *MBCP* Mother Baby Care point, *FP* Family planning

## Discussion

We estimated the prevalence of modern contraceptive use and unmet need for modern methods of contraception among postpartum women living with HIV in Kabarole District of Uganda. We further examined the factors associated with modern contraceptive use among the women. We found that less than half of the women were using modern methods of contraception while a third had unmet need for the methods. In addition, use of modern methods was significantly higher among women who were counseled on family planning during ANC than among those who were not. Similarly, use was significantly higher among women who obtained methods through the care points and among those who made joint decisions regarding childbearing with their partners than among those who did not.

Use of modern methods by women in the study was lower than the estimates of between 58 and 62% found in other studies among PLWHIV in Uganda [7, 44, 45]. The lower use of modern methods among postpartum women living with HIV compared to PLWHIV in general could be due to the unique conditions posed by the postpartum period, such as duration of postpartum amenorrhea [46], which could influence sexual and fertility behaviors of women, including whether or when to use contraception.

Injectables were the most used modern method among the postpartum women living with HIV. This is consistent with the country’s overall method mix where injectables are the most commonly used method [24]. There are several possible reasons for use of injectables: they may be convenient to use, women may consider them very effective at preventing pregnancy, and they can be used discretely in situations where the male partner is opposed to contraception. Due to the uncertainty of the efficacy of implants when one is on certain ART combinations [47, 48] and the HIV risk, drug interaction associated with injectable [49, 50], the high proportion of women choosing the two methods could be indicative of a need for greater counseling on dual protection and ensuring availability of a wide range of methods for the women.

Unmet need for modern contraception among women in the study was higher than the national average (28%) [51], but similar to unmet need among PLWHIV (38%) in Uganda [45]. The finding suggests that there are still missed opportunities for family planning service provision at the mother-baby care points despite integration of services at such outlets. Thus, even when postpartum women have numerous encounters with healthcare providers, they are unable to receive timely family planning services. Some of the women may never return to the facility, while others may return when they have conceived. The sub-optimal use of modern contraception and the prevailing unmet need among postpartum women who frequently interact with service providers are an indication of health systems challenges that may undermine Uganda’s ability to meet its target of reducing unmet need for contraception to less than 10% by 2020 [52].

Use of postpartum contraception generally increased with time since childbirth. Low use of contraception among women in the immediate postpartum period is consistent with findings from other studies [21]. This could be due to the low perceived risk to pregnancy often attached to the postpartum period [17]. Women who jointly made decisions regarding childbearing with their partners were significantly more likely to use modern contraceptive methods than those who did not jointly make such decisions. Literature shows that inter-spousal communication regarding childbearing enhances male involvement in family planning [32]. A qualitative study in Uganda, showed that one of the reasons why male partners were reluctant to discuss or provide permission to their spouses to use contraceptives was their desire for more children than the number desired by their spouses [53]. Possible policy initiatives to improve male partner involvement in family planning could include promotion of spousal agreement regarding fertility desire especially through better inter-spousal communication on how to achieve a desired family size. Moreover, since men play a significant role in the decision making concerning fertility, the needs for family planning should be made clear to them and males should be adequately informed on fertility and population issues.

Women who desired more children were significantly less likely to use modern contraceptives. The finding is consistent with those from demographic and health surveys in developing countries, including Uganda, which show that contraceptive use among postpartum women was associated with achievement of desired family size and current fertility desires [54]. In particular, postpartum women who had achieved their desired family size were more likely to use contraception than those who had not [54]. Although postpartum women who have not achieved their desired family size may be less inclined to use contraception because of the desire for more children, those living with HIV require appropriate counseling and guidance to reduce the risk of mother-to-child transmission of the disease [55]. The findings of significant association between use of a modern method and counseling on family planning during ANC as well as obtaining the methods through the care points suggest that integration of services is one way of improving postpartum contraceptive uptake.

The study findings may be influenced by certain limitations. First, participants were postpartum women enrolled in care at mother-baby care points. They may represent a select group of women, that is, those who have overcome certain barriers to accessing care. The findings may not be generalizable to other postpartum women living with HIV who are not enrolled into care. Due to the cross-sectional nature of the study, we cannot infer causality from the data and geographically, the study was in one district in Uganda, hence results may not be generalizable to other districts. Besides, there is potential for social desirability bias associated with self-reports that may affect the accuracy of measurements.

## Conclusion

Use of modern contraceptive methods was low while unmet need was high among postpartum women living with HIV who participated in the study. In spite of integration of services at the mother baby care points, our findings suggest the need for further strengthening of contraceptive service delivery at these HIV care points. Improved access to modern contraceptive methods at the care points, provision of counselling and promotion of male partner involvement through couple communication may enhance modern contraceptive use among postpartum women living with HIV. Providing early family planning counseling, even when women are still amenorrhoeic may improve uptake of contraception upon resumption of menses and sexual activity.

## Data Availability

The authors are pleased to share the dataset upon receiving request. All data to be shared will be de-identified and in Microsoft excel format. Interested parties may contact the corresponding author.

## Abbreviations

ANC: Antenatal Care
ART: Antiretroviral Therapy
PLWHIV: Persons Living with HIV
PR: Prevalence Ratio
MBCP: Mother Baby Care Point
